# American football: Watch Your Head!

**DOI:** 10.3389/frym.2021.652519

**Published:** 2021-10-21

**Authors:** Kelly Sarmiento, Dana Waltzman

**Affiliations:** 1 Division of Injury Prevention, National Center for Injury Prevention and Control, Centers for Disease Control and Prevention, Atlanta, GA, USA

## Abstract

Head impacts in American football may lead to brain injuries called concussions. To study head impacts in young people who play American football, we collected data using sensors in mouthguards worn by young American football players. The sensors counted the number of hits and bumps to the head (head impacts) that players of American tackle and flag football got during the football season. We found that tackle football players had about 15 times more head impacts during a game or practice than flag football players had, and 23 times more hard head impacts. Learning more about head impacts in young American football players can help scientists find ways to lower the chances of concussions and other injuries. That way, kids can enjoy the benefits of sports while keeping their brains safe.

## WHAT IS A CONCUSSION?

**Figure 1:**
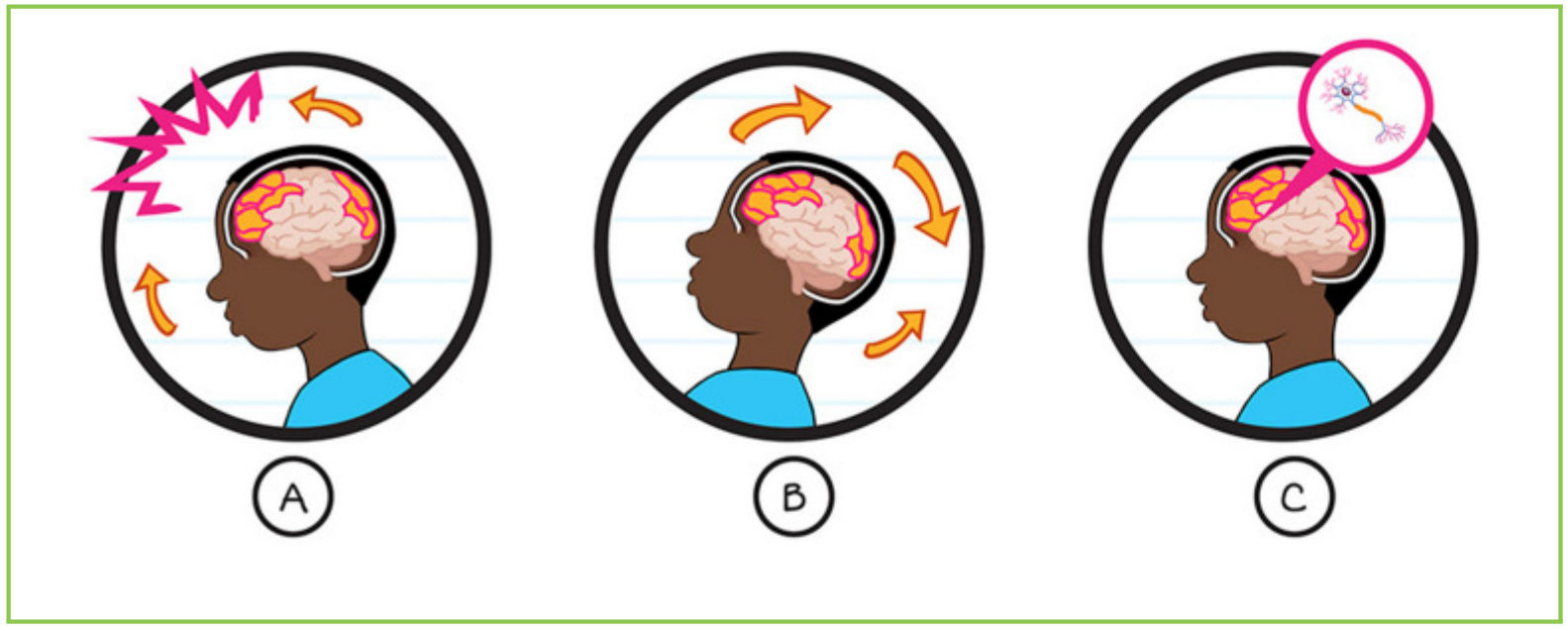
A concussion is a type of traumatic brain injury from a bump, blow, or jolt to the head or body. **(A)** When there is a jolt to the head or body, the head and brain move quickly back and forth. **(B)** The brain bounces and/or twists in the skull from the sudden movement. **(C)** Concussions can cause chemical changes in the brain’s cells, as well as stretching or other damage to the brain’s tissues.

You have probably heard about professional athletes getting **concussions** while playing sports. You may even know someone who has had a concussion, or maybe you have even had one yourself. If so, you may already know that a concussion is a brain injury (Figure 1). Concussions can change the way a person thinks, acts, feels, or learns during recovery [1]. For example, some kids get headaches, feel sick to their stomachs, or feel like they are in a fog while they are recovering from concussions. Others may have trouble concentrating and feel more tired than usual, making it hard to wake up for school.

During recovery, kids with concussions may need extra help at school for a short time [2]. They may also need to take a break from activities that can increase the chances of another head or brain injury, like playing sports or riding a bike [2]. Most kids have good recoveries and can get back to their regular activities within a couple of weeks after the concussion [2]. However, scientists have found that people with repeated concussions may experience longer recovery times or more severe symptoms [3].

Scientists are also trying to learn more about the long-term effects on the brains of people who experience multiple or repeated head impacts. Repeated head impacts include not only those that lead to concussions, but also impacts that *do not* cause the person to feel symptoms. Collisions experienced while playing sports are one way a person may receive repeated head impacts. There is a lot we do not know about head impacts that *do not* cause a person to feel symptoms. For example, we do not know whether these head impacts affect the brains of young athletes over time.

## HOW DO SCIENTISTS STUDY HEAD IMPACTS?

**Figure 2:**
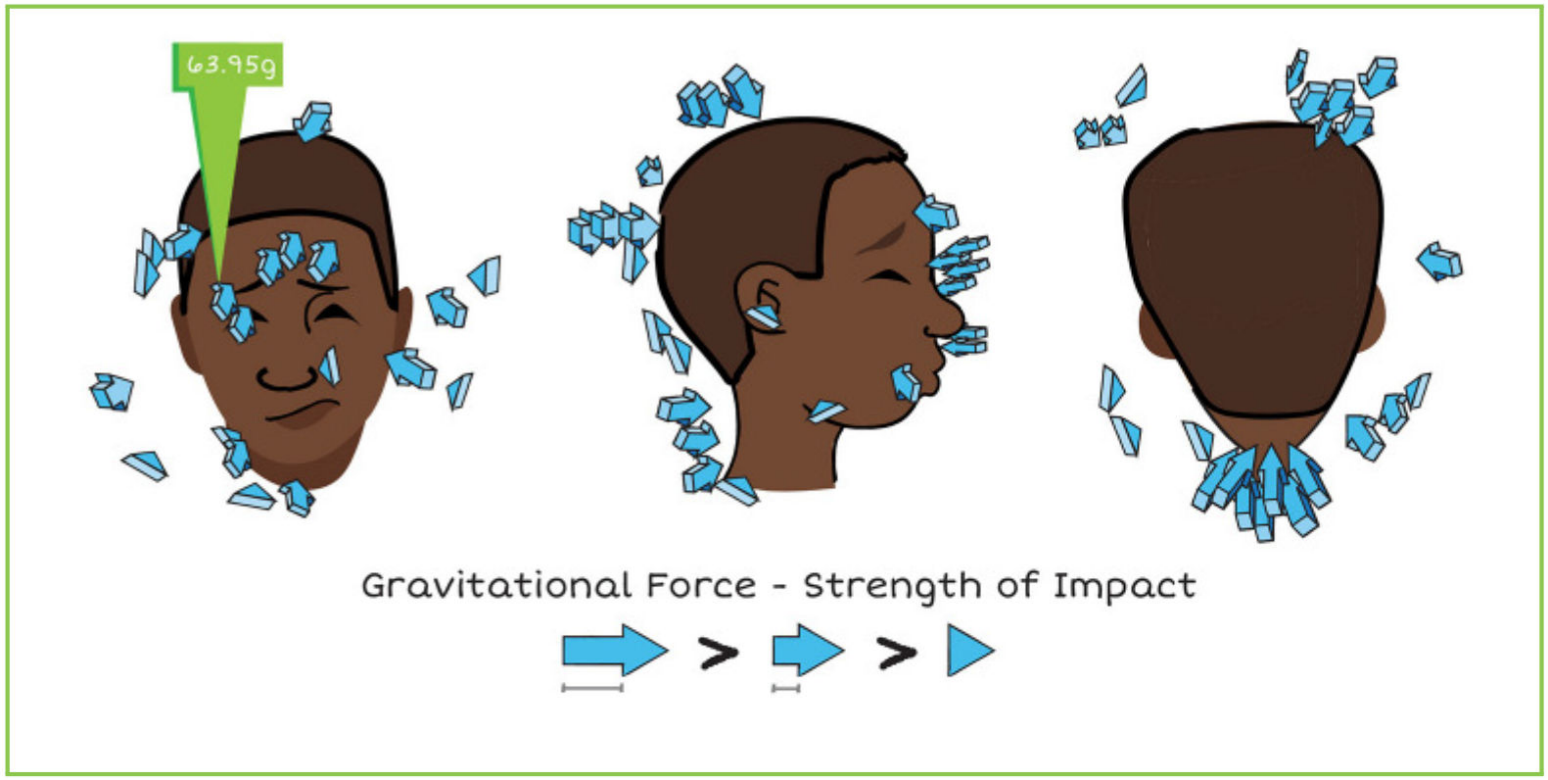
Data collected from an American football helmet sensor worn by a college player during two practice sessions. Each arrow shows where the player got a head impact and how strong the impact was. The longer the arrow, the stronger the impact. The strength of head impacts is measured in g-force. One impact, which was 63.95g, caused a concussion (image credit: Matthew Gfeller Sport-Related TBI Research Center, University of North Carolina at Chapel Hill).

To learn more about head impacts, scientists created tiny sensors. There are many types of sensors. Some were designed to be placed in a mouthguard or in an American football helmet. Others were created to fit in a patch that can be placed like a sticker on the side of an athlete’s head. The sensors keep count of each time an athlete gets a hit or bump to the head. The sensors can also tell whether the hit made the athlete’s head move front-to-back or side-to-side, or if the head twisted (Figure 2). However, sensors cannot tell if an athlete got a concussion from the impact.

**Figure 3:**
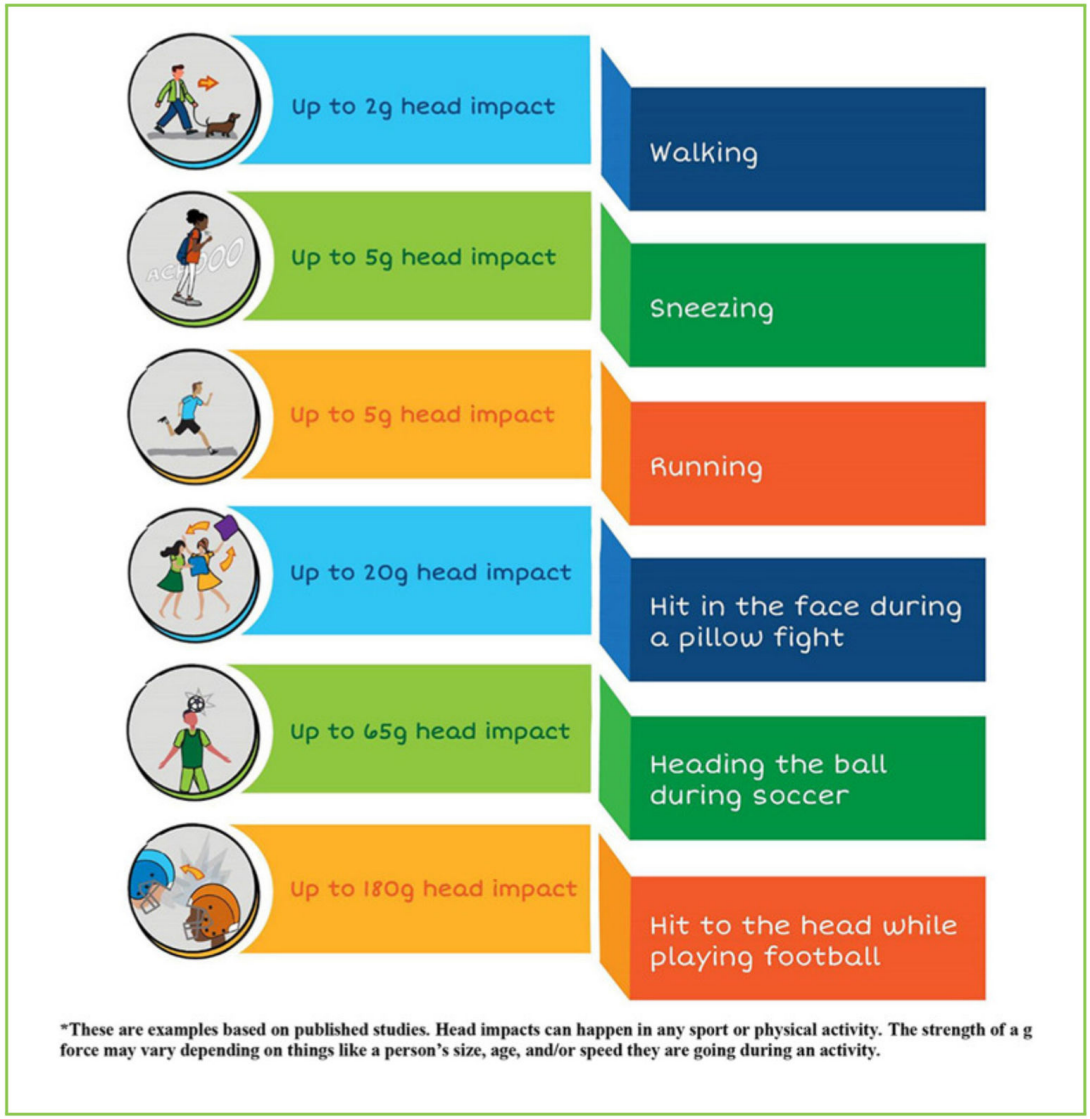
Examples of head impact g forces for different activities*.

Importantly, scientists also use sensors to measure the strength of each head impact, meaning whether an athlete got a hard or soft hit to the head. The sensor reports the strength of the head impact as a **g-force**, which stands for “gravitational force equivalent.” We may think of g-forces as what astronauts feel when they are launched into space. However, we experience g-forces to our brains many times during a normal day. When you are sitting still, you are at 1g. When you move your head, the g-force changes. For example, when your head moves when you sneeze or walk, you experience about a 3g head impact. Running or jumping is about a 5g head impact. Getting a hit to the face during a pillow fight may be as high as a 20g head impact. A hit while playing American football can be as high as a 180g head impact (Figure 3) [4].

An impact of 40g or higher increases the chance for concussion or other serious injury. But scientists do not currently know exactly how much g-force is needed to cause a concussion. Some people get concussions following a soft hit with a low g-force. Others do not get concussions after a hard, high g-force head impact.

## STUDYING HEAD IMPACTS IN YOUTH FOOTBALL

The excitement of catching the ball and running it into the endzone is just one reason why American football is one of the most popular sports played by young athletes in the United States. Each year, about 2 million kids take to the football field. While sports are a great way for kids to stay active, there is concern about head impacts and the safety of American football for young athletes. Tackle football is the most common type of American football played in the United States. Tackling involves knocking a player to the ground to end a play. Tackling is the biggest cause of head impacts and concussions in American football [5]. **Flag football** is a form of American football in which tackling is not allowed. Instead, an athlete removes a flag or flag belt from the ball carrier to end a play.

We wanted to learn how many head impacts a young American football player might get during a football season, depending on the type of American football played: tackle or flag. We gave mouthguards with sensors to over 540 players, ages 6–14 years. Some of the athletes played on tackle football teams and others played on flag football teams with no tackling allowed. Each athlete played with other players in the same age range. Athletes wore their sensor-containing mouthguards at every practice and game during the football season. At the end of the season, we gathered the data from the mouthguard sensors, which included information on the number of hits to the head and the g-force of each hit. We could also see whether the athlete got the head impact during a practice or a game.

## WHAT DID WE FIND?

From analyzing the sensor data, we found that tackle football players had about 15 times more head impacts (of any severity) than the flag football players during a game or practice [6, 7]. The tackle football players also had 23 times more *hard* head impacts than flag football players during a game or practice [6]. Both tackle and flag football players were more likely to get head impacts during games than during practice, but tackle football players got almost 20 times more head impacts during games than flag football players did [7].

## MAKING FOOTBALL SAFER FOR YOUNG ATHLETES

Our results clearly showed that tackle football players had a lot more head impacts than flag football players. There are several things that could be done to protect American football youth players from head impacts and concussions. For example, since fewer and less severe head impacts happen during flag football, increasing the number of flag football programs may allow more kids the option to play this version of the game. To make American tackle football safer, the number of drills that include tackling could be reduced. Rules or penalties could also be further enforced, which penalize athletes for hitting other players in the head. Scientists are also working to improve padding, playing surfaces, and American football helmets, to help protect athletes from injuries. Because the brain sits in fluid inside the skull, American football helmets cannot prevent the brain’s movement within the skull after a hit to the head, so these helmets cannot prevent all concussions. Still, an American football helmet that fits well can help protect a player from a skull fracture and lessen the damage to the brain from a hard hit to the head.

Learning more about head impacts in American football can help scientists find out how they may affect a young athlete’s brain. This knowledge can also help parents and leaders of sports programs learn the best ways to lower the chances of concussions and other injuries. That way, kids can enjoy the benefits of sports while keeping their brains safe!

## CONCUSSION

A type of brain injury from a bump, blow, or jolt to the head or body that leads to chemical changes in the brain that may affect how a person thinks, acts, feels, or learns during recovery.

## G-FORCE

(Gravitational force equivalent): The force that pulls or attracts all physical objects toward the center of our planet.

## FLAG FOOTBALL

A form of American football in which tackling is not allowed. Instead, an athlete removes a flag or flag belt from the ball carrier to end a play.
